# Notch signaling regulates pulmonary fibrosis

**DOI:** 10.3389/fcell.2024.1450038

**Published:** 2024-10-10

**Authors:** Xinyue Zhang, Zhihao Xu, Qi Chen, Zhimin Zhou

**Affiliations:** Department of Respiratory and Critical Care Medicine, The Fourth Affiliated Hospital, School of Medicine, Zhejiang University, Yiwu, China

**Keywords:** pulmonary fibrosis, notch, autophagy, mesenchymal transformation, macrophage polarization

## Abstract

Pulmonary fibrosis is a progressive interstitial lung disease associated with aging. The pathogenesis of pulmonary fibrosis remains unclear, however, alveolar epithelial cell injury, myofibroblast activation, and extracellular matrix (ECM) accumulation are recognized as key contributors. Moreover, recent studies have implicated cellular senescence, endothelial-mesenchymal transition (EndMT), and epigenetic modifications in the pathogenesis of fibrotic diseases. Various signaling pathways regulate pulmonary fibrosis, including the TGF-β, Notch, Wnt, Hedgehog, and mTOR pathways. Among these, the TGF-β pathway is extensively studied, while the Notch pathway has emerged as a recent research focus. The Notch pathway influences the fibrotic process by modulating immune cell differentiation (e.g., macrophages, lymphocytes), inhibiting autophagy, and promoting interstitial transformation. Consequently, inhibiting Notch signaling represents a promising approach to mitigating pulmonary fibrosis. In this review, we discuss the role of Notch signaling pathway in pulmonary fibrosis, aiming to offer insights for future therapeutic investigations.

## 1 Pulmonary fibrosis represents a central feature of interstitial lung diseases

Interstitial lung diseases (ILDs) are a heterogeneous group of respiratory diseases, which often result in varying degrees of pulmonary fibrosis and respiratory dysfunction. Despite numerous estimated causative factors, the etiology of ILDs remains unclear. Pulmonary fibrosis, a primary characteristic of ILD, arises from an exaggerated cascade of inflammatory and reparative responses within the pulmonary interstitium. This cascade, initiated by diverse disease factors, leads to structural remodeling of lung tissue,excessive deposition of extracellular matrix, and ultimately fibrosis formation (Distler et al., 2019). Fibrosis progresses to dyspnea and lung failure, often resulting in a high mortality rate. Idiopathic pulmonary fibrosis (IPF) represents the most prevalent form of fibrosing ILD. Clinically, patients typically present with respiratory symptoms, including cough and exertional dyspnea, which progress to deterioration of lung function. Imaging and pathological findings closely resemble those of usual interstitial pneumonia (UIP) (Inui et al., 2021). IPF primarily affects middle-aged and older adults, typically manifesting in the sixth and seventh decades of life, with an incidence that escalates significantly with age (King et al., 2011; Raghu et al., 2011; Wijsenbeek and Cottin, 2020). Fatality rates are high, with historical data reporting a median survival of 2–3 years post-diagnosis, and recent evidence indicates no improvement in survival rates (Raghu et al., 2011; Strongman et al., 2018). Recognized pathobiological mechanisms in IPF encompass epithelial cell dysfunction, impaired host defense, T-cell depletion, fibroblast activation, oxidative stress, vascular remodeling, alternative macrophage activation, and aging (Wells et al., 2018). Interactions among these pathways may be crucial. However, due to the unclear specific pathogenesis of pulmonary fibrosis, clinical treatment remains challenging. Currently, the only approved antifibrotic drugs by the US Food and Drug Administration are pirfenidone and nintedanib. Pirfenidone has been demonstrated to inhibit transforming growth factor-beta (TGF-β)signaling, fibroblast growth factor-2(FGF-2), and interleukin-1 beta (IL-1β), whereas the mechanism of action of nintedanib remains unknown (Oku et al., 2008; Schaefer et al., 2011; Ma et al., 2018). Nonetheless, the efficacy of drug therapy is limited, and lung transplantation remains the primary treatment modality. Consequently, investigating the mechanism of pulmonary fibrosis development and actively seeking effective and reliable drug treatments have become focal points of research in this field.

## 2 The Notch signaling pathway plays a pivotal role in the regulation of pulmonary fibrosis

The Notch signaling pathway is highly conserved. This pathway is involved in various processes, such as cell proliferation, apoptosis, and epithelial-mesenchymal transition (EMT) (Kopan and Ilagan, 2009). It comprises the Notch receptor, Notch ligand and CSL. In mammals, there are four Notch receptors (Notch1-Notch4),two Jagged family ligands (Jag1 and Jag2),and three delta-like ligands (Dll1, Dll3, and Dll4) expressed *in vivo*. Notch receptors are type I transmembrane proteins that form heterodimers in their extracellular domain (ECN) and transmembrane/intracellular domain (NTM). In the normal lung, four Notch receptors and five ligands are expressed. Notch1 predominates inalveolar epithelial cells, whereas Notch3 is prominent in vascular endothelial cells. Notch ligands, also type I transmembrane proteins, feature an extracellular domain with a cysteine-rich DSL motif. This domain is crucial for the mutual recognition and functional interaction with Notch receptors. CSL, also known as CBF1 or RBP-J in mammals, Su (H) in flies, and Lagl in nematodes, acts as a transcription factor. It modulates transcription by binding to various promoters (Fiúza and Arias, 2007; Jubb et al., 2010).

Activation of the Notch signaling pathway involves a three-step process (van Tetering and Vooijs, 2011). Initially, the full-length receptor is synthesized in the endoplasmic reticulum (ER) and undergoes proteolytic cleavage (S1 cleavage) mediated by furin-like convertases while transiting through the Golgi complex, resulting in the formation of a processed heterodimer. Subsequent binding of the ligand to the receptor triggers the metalloprotease ADAM to execute a second cleavage (S2 cleavage), followed by endocytosis of the remaining transmembrane and intracellular regions into recipient cells. The final cleavage (S3 cleavage) is carried out by γ-secretase, leading to the release of the Notch intracellular domain (NICD). NICD translocates into the nucleus where it interacts with the transcription factor recombination signal binding protein -J (RBP-J), recruiting other transcriptional regulation-related molecules to form transcriptional activation complexes that promote the expression of various downstream target genes, including Hey and Hes. The Hes family, predominantly expressed in mammals, with Hes1 and Hes5 being primary targets of Notch regulation. Similarly, the Hey family serves as downstream effectors of Notch signaling and is governed by the Notch pathway. These genes encode basic helix-loop-helix transcription factors, typically functioning as transcriptional repressors (Fiddes et al., 2018; Lim et al., 2019).

**FIGURE 1:**
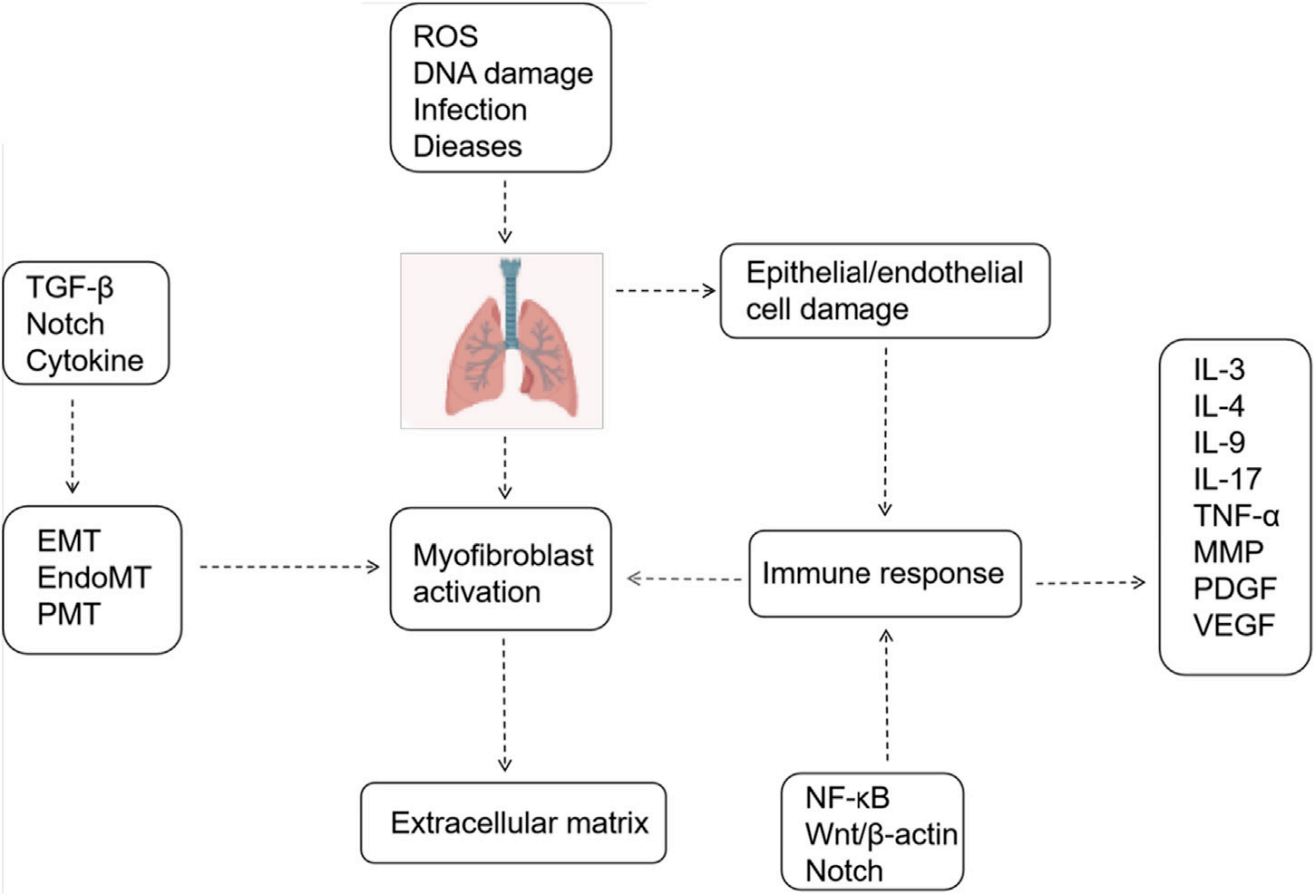
Mechanisms Associated with Pulmonary Fibrosis. Epithelial/endothelial cell injury, extracellular matrix deposition, and myofibroblast activation constitute three pivotal processes in pulmonary fibrosis. Various signaling pathways and cytokines regulate these processes.

The Notch signaling pathway plays a crucial role in lung development, with receptors and ligands expressed in both the lung epithelium and stroma. Recent studies have demonstrated the involvement of the Notch signaling pathway in lung fibrogenesis (van Tetering and Vooijs, 2011). The Notch signaling pathway-related protein expression is markedly upregulated in fibrotic alveolar regions of IPF patients (Aoyagi-Ikeda et al., 2011). Animal studies corroborate the activation of Notch signaling in pulmonary fibrosis, consistent with observations in lung specimens from patients with idiopathic interstitial pneumonia and pulmonary fibrosis (Liu et al., 2015). Researchers are actively investigating the role of Notch signaling in pulmonary fibrosis, particularly its interaction with the TGF-β pathway. Elevated TGF-β1 levels play a pivotal role in the IPF disease process, excessively activating TGF-β signaling, which induces myofibroblast differentiation and survival, enhances extracellular matrix production, inhibits matrix metalloproteinase-mediated matrix degradation, affects alveolar epithelial cells II(AEC II) to AEC I differentiation, and promotes cellular senescence (Thannickal et al., 2003; Thannickal and Horowitz, 2006; Bhaskaran et al., 2007; Horowitz et al., 2007). Notch1 can function as an effector gene of TGF-β. Activation of the TGF-β/Smad3 pathway facilitates Notch1 binding to its ligand Jagged-1, releasing NICD to translocate to the nucleus. There, it binds to the DNA-binding protein CSL, thereby upregulating downstream transcription factor HES-1 expression, enhancing extracellular matrix secretion and synthesis, and fostering pulmonary fibrosis development (Ostroukhova et al., 2006; Dees et al., 2011; Zhou et al., 2016). The Notch signaling pathway exhibits interactions with various other signaling pathways, facilitating pulmonary fibrosis progression. It synergizes with inflammatory signaling pathways such as nuclear factor kappa-B (NF-κB), mitogen-activated protein kinase (MAPK), and protein kinase B (Akt), thereby indirectly or directly enhancing the expression of factors like tumor necrosis factor-alpha (TNF-α) and TGF-β, thereby regulating fibrosis (Figure 1) (Wang et al., 2024). Recent studies have elucidated the multifaceted involvement of Notch signaling in pulmonary fibrosis. In a rat model of bleomycin-induced pulmonary fibrosis, the Jagged 1/Notch1 signaling pathway has been observed to induce alpha-smooth muscle actin (α-SMA) expression via NF-κB activation, promoting endothelial mesenchymal transition (EndMT) and accelerating fibrosis (Yin et al., 2018). Notch 1 additionally stimulates fibroplasia by inducing alveolar epithelial cell proliferation and inhibiting Napsin A and surfactant preprotein processing (Wasnick et al., 2023). Notch2 induces alterations in cellular expression of pro-fibrotic genes. and, notably, play a broader role in regulating basal cell maintenance and inhibiting ciliated cell differentiation, which are implicated in IPF pathogenesis (Lin et al., 2023). Notch3 promotes fibroblast survival by controlling myofibroblast differentiation and further regulates fibrosis by suppressing secretory primed basal (SPB) cells differentiation and secretory function (Carraro et al., 2020; Vera et al., 2021). Notch4 exacerbates pulmonary fibrosis by modulating pulmonary vascular endothelial cell differentiation and proliferation, leading to disrupted angiogenesis and lung tissue remodeling (Raymond et al., 2007). Therefore, precise modulation of inactive Notch signaling holds promise as a novel anti-fibrotic therapeutic approach.

## 3 The Notch signaling pathway contributes to pulmonary fibrosis through the regulation of immune cell differentiation

Although the precise etiology of idiopathic pulmonary fibrosis (IPF) remains elusive, both the innate and adaptive immune systems contribute significantly to its development (Wynn, 2011). Neutrophil elastase (NE), a secreted product of neutrophils, plays a critical role in promoting fibroblast proliferation, myofibroblast differentiation, and activating TGF-β, thereby contributing to pulmonary fibrosis (Gregory et al., 2015). Additionally, neutrophils contribute to fibrosis through cytokine release, secretion of proinflammatory factors, and generation of reactive oxygen species (ROS) (Zimmermann et al., 2010). Monocytes, circulating precursors of macrophages, become highly activated in fibrotic conditions and stimulate myofibroblast differentiation by releasing proinflammatory cytokine (Landsman et al., 2007; Zimmermann et al., 2010). Increased activation of dendritic cells (DCs) was observed in mouse lungs induced with bleomycin, with mitigation of pulmonary fibrosis noted upon prevention of their activation (Bantsimba-Malanda et al., 2010). Research indicates that alveolar macrophages exhibit both profibrotic and antifibrotic effects depending on their polarization, local microenvironment, and disease stage (Kolahian et al., 2016). Similarly, different T cell subsets also contribute significantly to the fibrotic process, with an imbalanced Th1/Th2 immune response considered pivotal in the pathogenesis of IPF (Heukels et al., 2019; Shenderov et al., 2021).

### 3.1 Macrophage polarization

Macrophages play a crucial role in both inflammation and host defense against invading pathogens. They exhibit considerable diversity and plasticity, delineated into two polarized states: M1 and M2, based on distinct activation modes and functions (Mills et al., 2000). M1 macrophages predominantly activate in response to Interferon-gamma (IFN-γ) and lipopolysaccharide, releasing pro-inflammatory mediators like IL-12, ROS, TNF-α, and IL-1, whereas M2 macrophages primarily respond to IL-4/IL-13, secreting anti-inflammatory agents such as IL-10, IL-13, and TGF-β(Gordon, 2003; Gordon and Martinez, 2010). Notably, the balance between IL12 and IL10 production critically distinguishes M1 from M2 macrophages (Martinez et al., 2008). Both M1 and M2 subsets contribute to pulmonary fibrosis pathogenesis, making modulating macrophage activation a promising therapeutic strategy. Initially, M1 macrophages drive the early inflammatory phase of pulmonary fibrosis by inducing inducible nitric oxide synthase (INOS) to generate NO and releasing pro-inflammatory cytokines, causing damage to alveolar and vascular epithelial cells. As pulmonary fibrosis advances, M1 macrophages transition into M2 macrophages, secreting IL-10, IL-13, TGF-β, among others, to mediate the anti-inflammatory and immunosuppressive phases. However, the excessive accumulation of M2 macrophages in lung tissue leads to heightened expression of pro-fibrotic factors like TGF-β, vascular endothelial growth factors (VEGF), platelet-derived growth factor (PDGF), and Th2 cytokines including IL-13 and IL-33, exacerbating pulmonary fibrosis development (Li et al., 2014; Zhang et al., 2022).

Macrophage differentiation is a dynamic process characterized by the ability to transition between various phenotypes as required (Stout et al., 2005; Biswas and Mantovani, 2010). Various signaling pathways, including the signal transducer and activator of transcription (STAT) proteins STAT 1 and NF-κB, contribute to macrophage polarization. Specifically, STAT 1 and NF-κB are involved in the activation of M1 phenotype, leading to the generation of cytotoxic and inflammatory cells, while STAT 3/6 regulates M2 phenotype, which is associated with immunosuppressive and tumor-promoting activities (Pagie et al., 2018). Recently, some studies revealed that Notch signaling plays a pivotal role as an endogenous mechanism in determining the polarization of M1 and M2 phenotypes during macrophage activation (Wang et al., 2010). Overexpression of Notch signaling induces the expression of the M1 phenotype in macrophages, whereas inhibition of Notch signaling leads to the expression of the M2 phenotype (Wang et al., 2010). Research suggests that inhibiting the Notch signaling pathway to regulate macrophage polarization may improve pulmonary fibrosis and facilitate lung function recovery (Wang et al., 2023). However,the precise mechanism through which Notch regulates lung macrophage polarization remains elusive. Notch signaling can be triggered by the Toll-like receptor (TLR) signaling stimulation and subsequently regulated within macrophages (Palaga et al., 2007). Specifically, RBP-J enhances the TLR4-induced expression of key mediators in M1 macrophages (Xu et al., 2012). Additionally, studies suggest that Dll4 may be pivotal in this process. Dll4 has been shown to promote blood monocyte polarization towards M1 type cells while inhibiting IL-4-induced M2 type polarization. Notably, heightened Dll4 expression induces apoptosis in M2 cells (Pagie et al., 2018). The NICD also interacts with hypoxia-inducible factor (HIF-α), contributing to M1 activation by regulating sugar fermentation turnover (Landor et al., 2011). Additionally, evidence suggests that Notch signaling predominantly regulates macrophage polarization via the suppressor of cytokine signaling (SOCS3). The SOCS family comprises inducible inhibitors of cytokine signaling, pivotal in constraining inflammatory responses (Dalpke et al., 2008). Within macrophages and dendritic cells, SOCS proteins regulate not only cellular sensitivity to cytokines but also signaling through Toll-like receptors (TLR). Unique expression of SOCS3 is indispensable for classical macrophage activation (Liu et al., 2008).

### 3.2 T-cell differentiation

**FIGURE 2:**
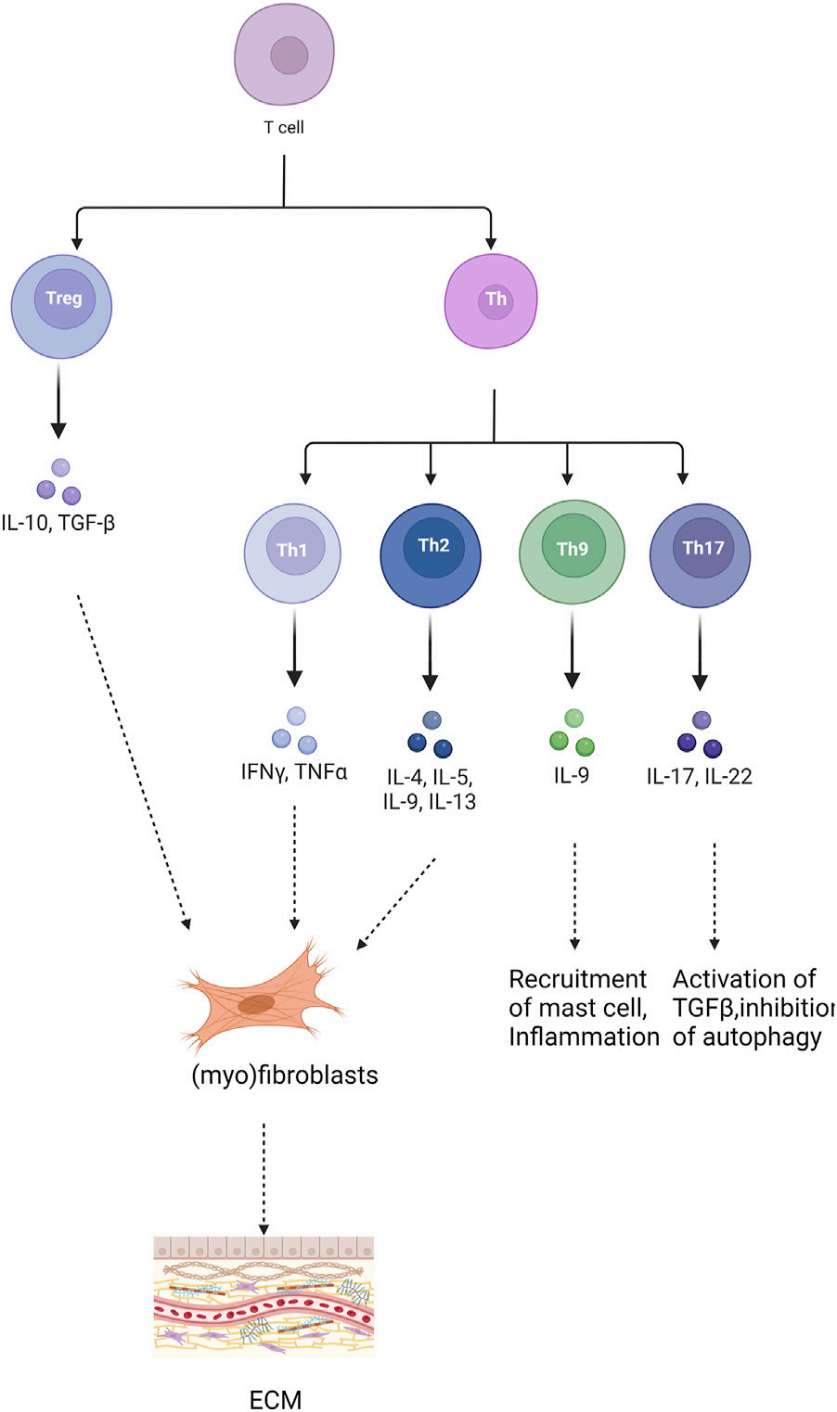
Role of different T cells in pulmonary fibrosis. “+” represents “activation”; “−” represents “inhibition”.

T cells play a significant role in pulmonary fibrosis through various subsets and the release of inflammatory cytokines. Inhibiting the activation of inflammatory factors can mitigate immune damage and inflammatory responses, thereby achieving anti-fibrotic effects and delaying the onset and progression of pulmonary fibrosis (Jee et al., 2019). Differentiated by their distinct pathways in inflammation and immunity, T cells encompass various subsets, including CD4^+^ T helper lymphocytes (Th), regulatory T cells (Treg), CD8^+^ cytotoxic T cells (Tc), and natural killer T cells (NKT). These diverse T lymphocyte populations profoundly influence the initiation and advancement of pulmonary fibrosis, exerting varying degrees of impact on the fibrotic process (Figure 2). Th1 cells secrete cytokines such as IFN-γ to mitigate lung tissue injury and retard pulmonary fibrosis progression by diminishing fibroblast activity, collagen production, and extracellular matrix deposition (Liu et al., 2002). Conversely, Th2 cells produce an array of inflammatory factors, including IL-4, IL-13, IL-31, and TGF-β, which foster fibroblast and macrophage activation, as well as collagen synthesis, thereby promoting fibrogenesis (Sempowski et al., 1996; Zhou et al., 1999). Th9 cells expedite fibrosis progression by releasing pro-inflammatory cytokines like IL-4, stimulating mast cells to secrete TGF-β, and inhibiting the expression of anti-fibrotic cytokines such as IFN-γ(Noelle and Nowak, 2010; Overed-Sayer et al., 2014; Deng et al., 2021). Th17 cells bolster pulmonary fibrosis development and advancement by fostering collagen secretion and extracellular matrix accumulation through their secretion of IL-17 (Zhu and Qian, 2012). Regulatory Treg cells accelerate pulmonary fibrosis by secreting profibrotic cytokines like TGF-β, disrupting the Th1/Th2 balance (Wang et al., 2020). Additionally, they foster pulmonary fibrosis by suppressing Th1 cytokine secretion while enhancing Th2 cytokine secretion (Zhang et al., 2020). Conversely, Treg cells facilitate epithelial cell repair, impede fibroblast accumulation, and decrease the production of pro-inflammatory factors, thereby inhibiting pulmonary fibrosis (Wang et al., 2020). Notably, Th1/Th2 cells, the predominant T cell subset in immune inflammation, exert significant effects on pulmonary fibrosis, making the regulation of their balance a current research focus. Numerous studies have implicated Notch signaling in modulating Th1 and Th2 responses (Zhao et al., 2019; Wu et al., 2023). Inhibition of Notch signaling significantly reduced T cell proliferation. Additionally, it reversed the predominant Th2 inflammation and increased the Th1 ratio when the Notch signaling pathway was inhibited using DAPT (Adler et al., 2003). Moreover, Notch signaling inhibition decreased the proportion of Th2 cells and subsequently attenuated fibrosis in a rat model of pulmonary fibrosis in peripheral blood lymphocytes (PBL) (Li et al., 2023). Activation of various receptors and ligands within the Notch signaling pathway exert distinct effects on CD4^+^ T cell differentiation. Specifically, Dll1 induces Th1 cell differentiation, whereas Jagged induces Th2 cell differentiation (Amsen et al., 2015; Nouri-Shirazi et al., 2015). Research findings using rat models of asthma demonstrated that the presence of Notch1 and Notch2 on CD4^+^ T cells, along with Jagged1 on dendritic cells and/or Dll1 on smooth muscle cells, facilitated asthma development. Conversely, interaction with Dll4 expressed on dendritic cells inhibited both airway inflammation and hyperreactivity (Lafkas et al., 2015).

## 4 The Notch signaling pathway inhibits autophagy and plays a role in pulmonary fibrosis

Autophagy is a catabolic process crucial for maintaining cellular homeostasis through various mechanisms, including autophagosome formation, transport, and lysosomal degradation of long-lived proteins, damaged organelles, and intracellular pathogens. Autophagy is categorized into selective and non-selective forms. While typically non-selective, in pathological states, it can selectively isolate intracellular aberrant components for degradation (Sosulski et al., 2015). Prior research indicates diminished autophagic activity in lung fibroblasts from patients with IPF and demonstrates reduced levels of Beclin1 expression alongside elevated p62 expression in mice induced with bleomycin (Romero et al., 2016; Krempaska et al., 2020). Notably, p62 serves as a substrate for selective autophagy. During the initiation of autophagy, p62 proteins can bind to ubiquitinated abnormally folded protein aggregates in the cytoplasm. Subsequently, these p62-bound ubiquitinated protein aggregates are degraded and eliminated through autophagosome-lysosome fusion following receptor recognition (Katsuragi et al., 2015). Additionally, Beclin1 serves as a principal regulator of autophagy. The induction of autophagy correlates closely with heightened Beclin1 expression, facilitating the recruitment of proteins crucial for initiating autophagosome formation (Vishnupriya et al., 2020; Huang et al., 2021). The mechanisms underlying pulmonary fibrosis via autophagy may involve the inhibition of ROS production, fibroblast proliferation and differentiation, epithelial cell senescence, and EMT (Li et al., 2023). Insufficient autophagy may expedite fibroblast differentiation into myofibroblasts and promote p21-mediated senescence of lung epithelial cells (Eickelberg et al., 2012; Ashour et al., 2020).

**FIGURE 3:**
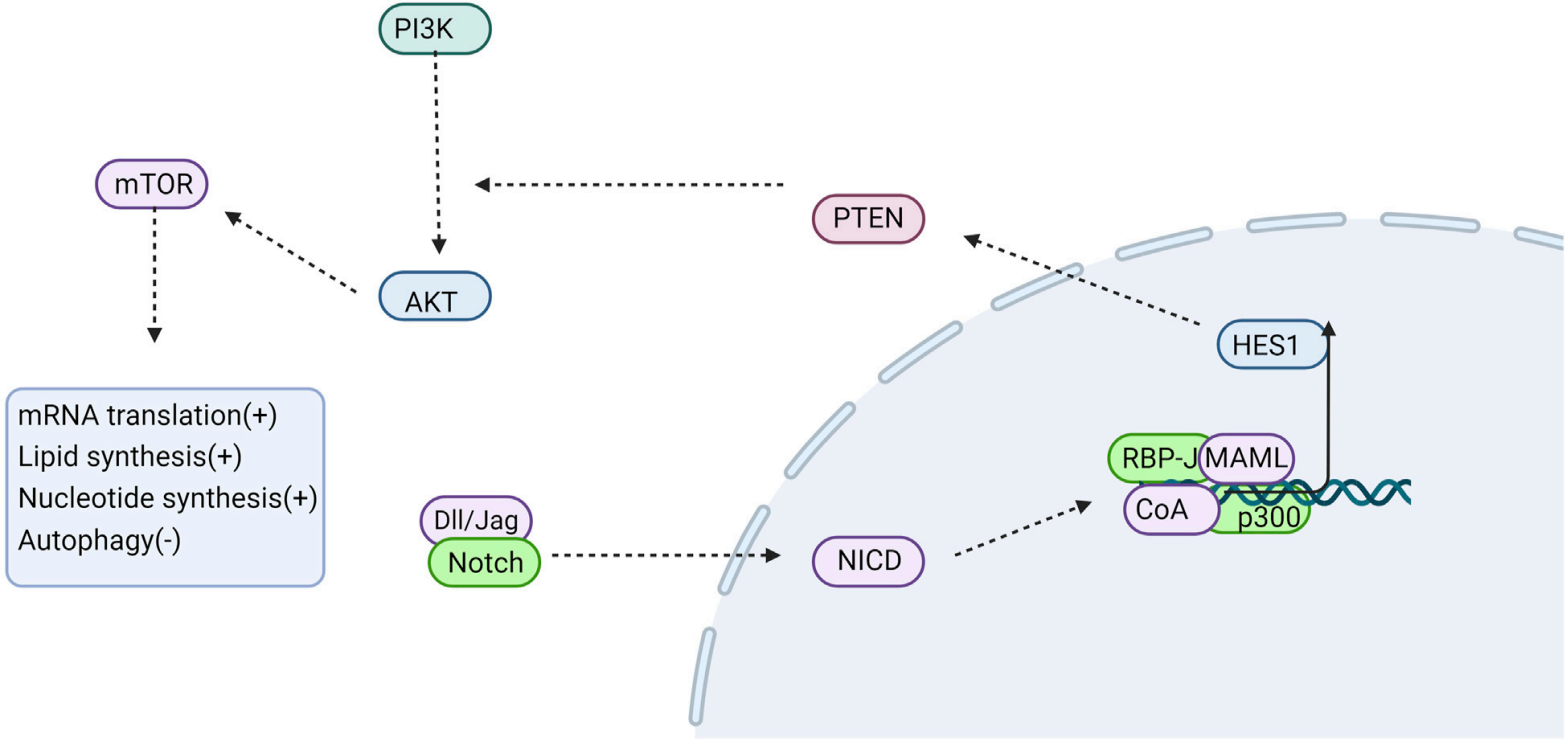
Notch signaling is involved in regulating autophagy. NOTCH down-regulates PTEN protein expression via its downstream target gene HES1. HES1 subsequently activates PI3K, which in turn phosphorylates AKt, thereby transmitting signals to various downstream effectors including mTOR. Phosphorylated mTOR further phosphorylates downstream effector molecules, thus promoting protein synthesis and inhibiting autophagy. CoA, coactivator; MAML, Mastermind-like; “+” represents “activation”; “−” represents “inhibition”.

Autophagy is regulated by various factors and signaling pathways, with reported associations with Notch signaling pathways across different cell models. In directed stem cell differentiation, Notch signaling can interact with autophagy, with the inhibition of Notch signaling down-regulating the expression of autophagy-related protein LC3 and the production of inflammatory factors INF-γ, TNF-α, and IL-1β(Wu et al., 2016). Autophagy can facilitate the cardiac differentiation of P19CL6 cells by eliminating the NICD(Jia et al., 2014). Activated NLRP3 inflammasomes can induce myofibroblast differentiation, whereas autophagy may attenuate fibrosis progression by suppressing NLRP3 inflammasome activation (Ding et al., 2021). Research indicates that autophagy modulates NLRP3 inflammasome activity via the Notch1 signaling pathway (Ji et al., 2021). Therefore, it is hypothesized that the Notch pathway influences lung fibrosis through its regulation of autophagy. The PI3K/Akt/mTOR signaling pathway is a extensively studied axis associated with autophagy regulation (Yu et al., 2019). PI3K activates Akt, which in turn signals to several downstream effectors, including mTOR. Phosphorylation of downstream effector molecules by mTOR promotes protein production while inhibiting autophagy (Yan et al., 2014). Activated Phosphatase and Tensin Homolog deleted on Chromosome 10 (PTEN) lipid phosphatase activity has been shown to regulate autophagy, cell proliferation, and apoptosis by negatively modulating the PI3K/Akt signaling pathway. In research on tubulointerstitial fibrosis associated with diabetic nephropathy, it was observed that Notch1 downregulates PTEN protein expression via the transcription factor Hes1. This downregulation results in reduced autophagy levels and increased extracellular matrix protein synthesis in tubular epithelial cells, thereby promoting the development and progression of renal interstitial fibrosis (Liu et al., 2018). Similarly, studies on breast cancer have shown that HES1 inhibits the expression of the tumor suppressor gene P53 and prevents apoptosis through the PTEN/PI3K/AKT/mTOR signaling pathway (Pappas et al., 2021). Furthermore, it has been found that inhibition of the Notch signaling pathway can regulate autophagy in mesenchymal stem cells via the PTEN/PI3K/Akt/mTOR pathway (Song et al., 2015). Therefore, it is plausible to speculate that the Notch signaling pathway regulates autophagy through the PTEN/PI3K/AKT pathway (Figure 3).

## 5 Notch signaling induces mesenchymal transformation and contributes to pulmonary fibrosis progression

The activation and proliferation of myofibroblasts are crucial processes in the pathogenesis of pulmonary fibrosis (Peng et al., 2020). These activated cells produce an excess of extracellular matrix, leading to structural remodeling of lung tissue (Spagnolo et al., 2021). Research indicates that myofibroblasts in pulmonary fibrosis originate from various sources, including tissue-resident lung mesenchymal cells, epithelial cells, local fibroblasts, pericytes, circulating fibrocytes, and progenitor stem cells derived from both circulating and bone marrow. Mesenchymal transition is a crucial mechanism giving rise to myofibroblasts. This process encompasses epithelial-mesenchymal transition, endothelial-mesenchymal transition, and pericyte-mesenchymal transition (Inui et al., 2021).

### 5.1 Epithelial-mesenchymal transition

EMT is a pathological process in tissue remodeling, characterized by the loss of epithelial cell characteristics and acquisition of mesenchymal traits. During EMT, epithelial cell markers like E-cadherin and cytokeratin decrease, while mesenchymal markers such as vimentin, fibronectin, fibroblast-specific protein 1 (FSP-1), αSMA, and N-cadherin increase (Inui et al., 2021). EMT plays a crucial role in pulmonary fibrosis development. Immunohistochemical staining of lung tissue from patients with IPF demonstrated epithelial cells undergoing transformation into mesenchymal cells. Inhibition of the EMT process significantly ameliorated pulmonary fibrosis in mice induced by bleomycin (Peng et al., 2020). The EMT process is regulated by various cytokines and signaling pathways, notably TGF-β, Wnt, Notch, Hedgehog, and MAPK-dependent pathways. Among these, TGF-β is the most extensively studied inducer of EMT. TGF-β1 primarily regulates ECM secretion by activating its downstream Smad signaling pathway. This activation promotes phosphorylation of Smad2/3 and their binding to Smad4. Subsequently, the Smad complex translocates into the nucleus, ultimately influencing the development of EMT and progression of pulmonary fibrosis (Zhang et al., 2016). In recent years, there has been significant attention on the impact of Notch signaling on EMT. Studies have revealed that in bleomycin-induced pulmonary fibrosis, mice lacking Notch1 exhibit reduced levels of myofibroblasts and collagen I in the lung compared to controls. Moreover, inhibition of Notch1 using DAPT impedes the conversion of fibroblasts to myofibroblasts, resulting in the downregulation of vimentin and α-SMA protein expression, thereby impeding the progression of pulmonary fibrosis (Hu et al., 2015). The specific regulatory mechanism of Notch in EMT has been elucidated, suggesting that the Notch signaling pathway may directly regulate EMT via its intracellular domain mediation. For instance, studies have shown that embryos lacking expression of Notch1 or its transcriptional binding partner RBPJ fail to express snail, crucial in the EMT process, during cardiovascular system development (Dongre and Weinberg, 2019). Additionally, Notch indirectly regulates EMT by interacting with various signaling pathways. Notch1 has been found to induce alveolar epithelial cells to undergo mesenchymal transformation through the TGF-β1-Smad3 pathway. Furthermore, it can promote the transformation of fibroblasts into myofibroblasts, consequently leading to ECM formation and accumulation, ultimately contributing to pulmonary fibrosis development (Chen et al., 2013). Hence, Notch1 plays a pivotal role in EMT, myofibroblast differentiation, and collagen fiber formation during pulmonary fibrosis pathogenesis.

### 5.2 Endothelial-mesenchymal transition

Recent studies have demonstrated that endothelial cells represent an additional significant source of myofibroblasts (Hashimoto et al., 2010; Piera-Velazquez and Jimenez, 2012). EndMT is a specialized process akin to EMT, wherein endothelial cells undergo phenotypic changes marked by the loss of endothelial-specific markers (e.g., CD31, von Willebrand factor, and vascular endothelial cadherin), acquisition of mesenchymal markers, and disassembly of cell-cell junctions (Tian et al., 2021). A variety of transcription factors and signaling pathways are involved in the process of EndMT, and the main signaling pathways are TGF-β/Samd3, BMP, Notch, and Wnt signaling pathway, which cross each other and affect each other to promote the development of EndMT. Recently, EndMT has emerged as an important mechanism in the development of IPF, with signaling pathways such as Dll4/Notch4 and Jagged 1/Notch1 prominently implicated in its progression. Dll4 is expressed in vascular endothelial cells and triggers intracellular signaling upon binding to receptors like Notch1 and Notch4 on neighboring cells, regulating angiogenesis and development. In murine pulmonary fibrosis, activation of Dll4/Notch4/CBF1 signaling intensifies interstitial transformation by impeding endothelial cell proliferation (Raymond et al., 2007). In a separate investigation, researchers discovered activation of the Jagged 1/Notch 1 signaling pathway in pulmonary microvascular endothelial cells (PMVECs), promoting EndMT, a process playing multiple significant roles in the pathogenesis of pulmonary fibrosis (Yin et al., 2018). However, expression of Dll4 and Jagged 4 decreased during this progression. Therefore, elucidating changes in Notch 1 and Notch 4 signaling pathways in lung fibrosis, and their correlation with EndoMT, is crucial for halting further abnormal transdifferentiation and fibrotic lung disease progression, while preserving normal tissue remodeling. Notably, Notch signaling potentially synergizes with TGF-β in EndMT modulation. Specifically, Notch signaling regulates EndoMT by releasing NICD, up-regulating intracellular Smad3 mRNA, and stabilizing Smad3 protein, in conjunction with TGF-β (Liu et al., 2014). Moreover, Notch directly influences EndMT by modulating the expression of transcription factors Snail, Slug, and ZEB1, thereby upregulating Snail and Slug proteins while inhibiting endothelial cell adhesion molecule VE-cadherin expression, ultimately disrupting endothelial cell adhesion junctions and facilitating mesenchymal cell transition (Gasperini et al., 2012). Furthermore, synergy between the Notch signaling pathway and TGF-β on EndMT has been observed, with Notch signaling enhancing intracellular Smad3 mRNA levels via NICD release and stabilizing Smad3 protein, thereby co-regulating EndoMT with TGF-β (Liu et al., 2014).

### 5.3 Pericyte-mesenchymal transition

Pericytes constitute heterogeneous cell populations located in the perivascular space. In mouse lung tissue, pericytes expressing high levels of NG2 are found on arterioles, while those on venules lack NG2 expression. Pericytes associated with capillaries also express NG2, and all pericytes in the lung express desmin (Shammout and Johnson, 2019). Pericytes also express PDGFRβ, a receptor for the PDGF protein, on their surface (Johnson et al., 2015). It has been demonstrated using genetic cell labeling techniques that pericytes can act as precursors of myofibroblasts (Barron et al., 2016). Additionally, pericytes have been implicated in kidney and nervous system fibrosis (Lin et al., 2008). Pericytes play a physiological role in maintaining vascular homeostasis by regulating vascular tone, secreting ECM components, modulating leukocyte extravasation, and producing mediators essential for vascular homeostasis and angiogenesis (Shammout and Johnson, 2019). Nonetheless, under pathological conditions, PMT readily occurs. This process involves pericyte detachment from the endothelial cell wall, migration, and subsequent transformation into myofibroblasts, leading to collagen deposition and matrix remodeling, thereby promoting tissue fibrosis (Chang et al., 2012). PMT has been documented in numerous respiratory ailments, including allergic asthma and pulmonary hypertension (Shammout and Johnson, 2019). Recently, inhibiting pericyte differentiation into myofibroblasts has emerged as a potential therapeutic approach for ameliorating renal fibrosis associated with diabetic nephropathy (Chang et al., 2012). Consequently, modulating PMT holds promise as a treatment avenue for IPF.

It has been suggested that inflammatory stimuli and oxidative stress lead to TGF-β overproduction and separation of pericytes from endothelial cells, which leads to detachment, migration, and transdifferentiation of pericytes into myofibroblasts (Ballermann and Obeidat, 2014; Kulshrestha et al., 2020). Recent research indicates the involvement of Notch signaling in pericyte differentiation. Notch 1 induces differentiation into glioblastoma stem cells, Notch 3 supports pericyte population in cerebral vessels, and Dll 4 prompts differentiation into myofibroblasts in primary cultured renal pericytes (Wang et al., 2019). Notch signaling is widely implicated in pericyte differentiation based on available evidence, although the specific mechanism remains elusive. Studies using mouse models of pulmonary hypertension suggest that targeting the TGF-β/CXCR7 and CXCR4 signaling pathways can alleviate pericyte dysfunction in PAH. Additionally, Notch signaling may impact PMT by interacting with TGF-β. Another study focusing on IPF revealed that Notch1 regulates microangiogenesis by modulating PDGFRβ expression, a receptor present on pericytes. Notably, it may also regulate PMT via the PDGFRβ/ROCK 1 signaling pathway (Nouri-Shirazi et al., 2015; Wang et al., 2019).

## 6 Conclusion and perspetives

Pulmonary fibrosis, a prevalent lung ailment associated with substantial mortality rates, presents limited treatment options and efficacy. Two drugs, pirfenidone and nintedanib, are clinically approved for its treatment. Pirfenidone has been shown to inhibit TGF-β signaling, fibroblast growth factor-2, and IL-1β (Oku et al., 2008; Schaefer et al., 2011; Ma et al., 2018). In contrast, the exact mechanism of action of nintedanib remains unclear. Early studies indicated that nintedanib inhibits the phosphorylation of vascular endothelial growth factor receptor (VEGFR), platelet-derived growth factor receptor (PDGFR), and fibroblast growth factor receptor (FGFR), as well as the TGF-β pathway, thereby delaying pulmonary fibrosis (Chaudhary et al., 2007; Hostettler et al., 2014; Wollin et al., 2014; Rangarajan et al., 2016). Recent research has demonstrated that nintedanib also delays bleomycin-induced EMT and lung fibrosis by inhibiting the Src pathway (Li et al., 2017). Additionally, nintedanib has been found to inhibit Wnt3a-induced β-catenin nuclear translocation by blocking Src kinase activation and β-catenin phosphorylation, thus inhibiting Wnt/β-catenin signaling-induced fibrotic progression (Li et al., 2020).

However, the therapeutic efficacy of drugs is limited. Therefore, understanding the pathogenesis of pulmonary fibrosis is crucial for identifying effective therapeutic targets and improving prognosis. The triad of extracellular matrix deposition, myofibroblast proliferation, and alveolar epithelial cell injury constitutes the primary pathways in pulmonary fibrosis, thus emphasizing the significance of regulating these pivotal processes to impede disease progression. Recent investigations have elucidated a profound association between aging and pulmonary fibrosis, with autophagy deficiency emerging as a notable contributor to cellular senescence. Furthermore, cellular abnormalities significantly contribute to the accelerated differentiation of fibroblasts into myofibroblasts and the ensuing accumulation of extracellular matrix. Inflammatory cells, through the secretion of diverse cytokines and chemokines, foster pulmonary fibrosis via various pathways. Notably, macrophages and T cells wield considerable influence over fibrotic and anti-fibrotic processes, potentially ameliorating pulmonary fibrosis by modulating cellular differentiation patterns. Myofibroblast proliferation and differentiation stand as principal determinants of extracellular matrix deposition, with mesenchymal transformation serving as their primary source. While EMT traditionally garners attention in fibrotic disorders, recent scrutiny has extended to EndMT and PMT, with EndMT assuming a pivotal role in sustaining inflammation and collagen secretion. The Notch signaling pathway has been implicated in the pathogenesis of IPF. various findings demonstrate that the Notch signaling pathway primarily induces EMT, myofibroblast differentiation, and cellular senescence by cross-talking with various signaling cascades. Additionally, it directly modulates effector cells to enhance collagen secretion, induce autophagy dysfunction, and participate in other processes implicated in pulmonary fibrosis pathogenesis. Nevertheless, the precise mechanisms underlying the Notch signaling pathway’s involvement in pulmonary fibrosis remain elusive. By elucidating its regulatory mechanisms, we can enhance our understanding of pulmonary fibrosis pathogenesis, thereby identifying novel therapeutic targets.
