# Supplementary material for: Notch signaling regulates pulmonary fibrosis
Source: Front Cell Dev Biol. 2024 Oct 10;12:1450038. doi: 10.3389/fcell.2024.1450038 (PMC11499121; doi:10.3389/fcell.2024.1450038)
Supplement: Supplementary file 1 [file Table1.DOCX]

Supplementary Material

# Supplementary Figures

**
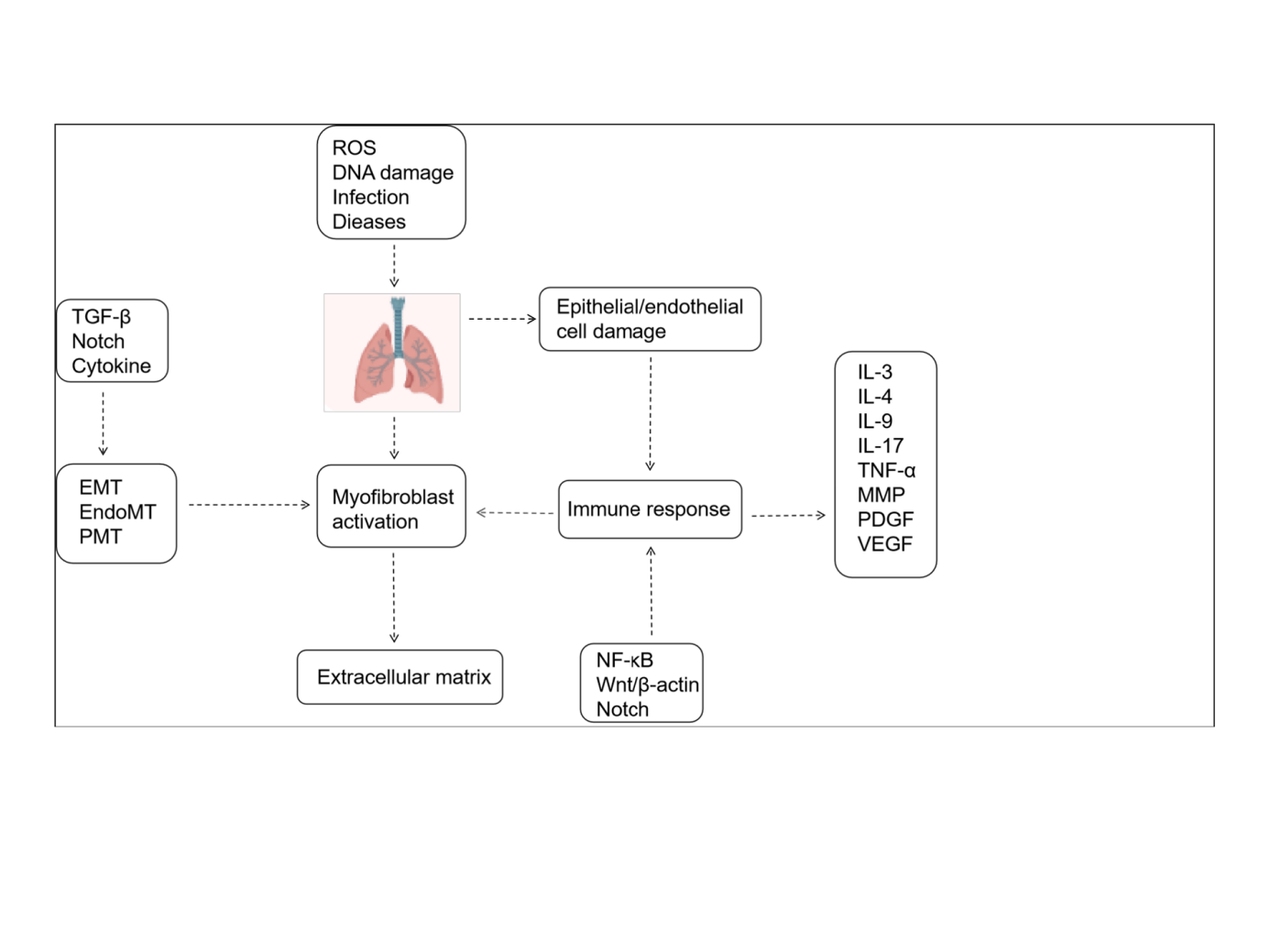
**

**Supplementary Figure 1.** Mechanisms Associated with Pulmonary Fibrosis.Epithelial/endothelial cell injury, extracellular matrix deposition, and myofibroblast activation constitute three pivotal processes in pulmonary fibrosis. Various signaling pathways and cytokines regulate these processes.

**
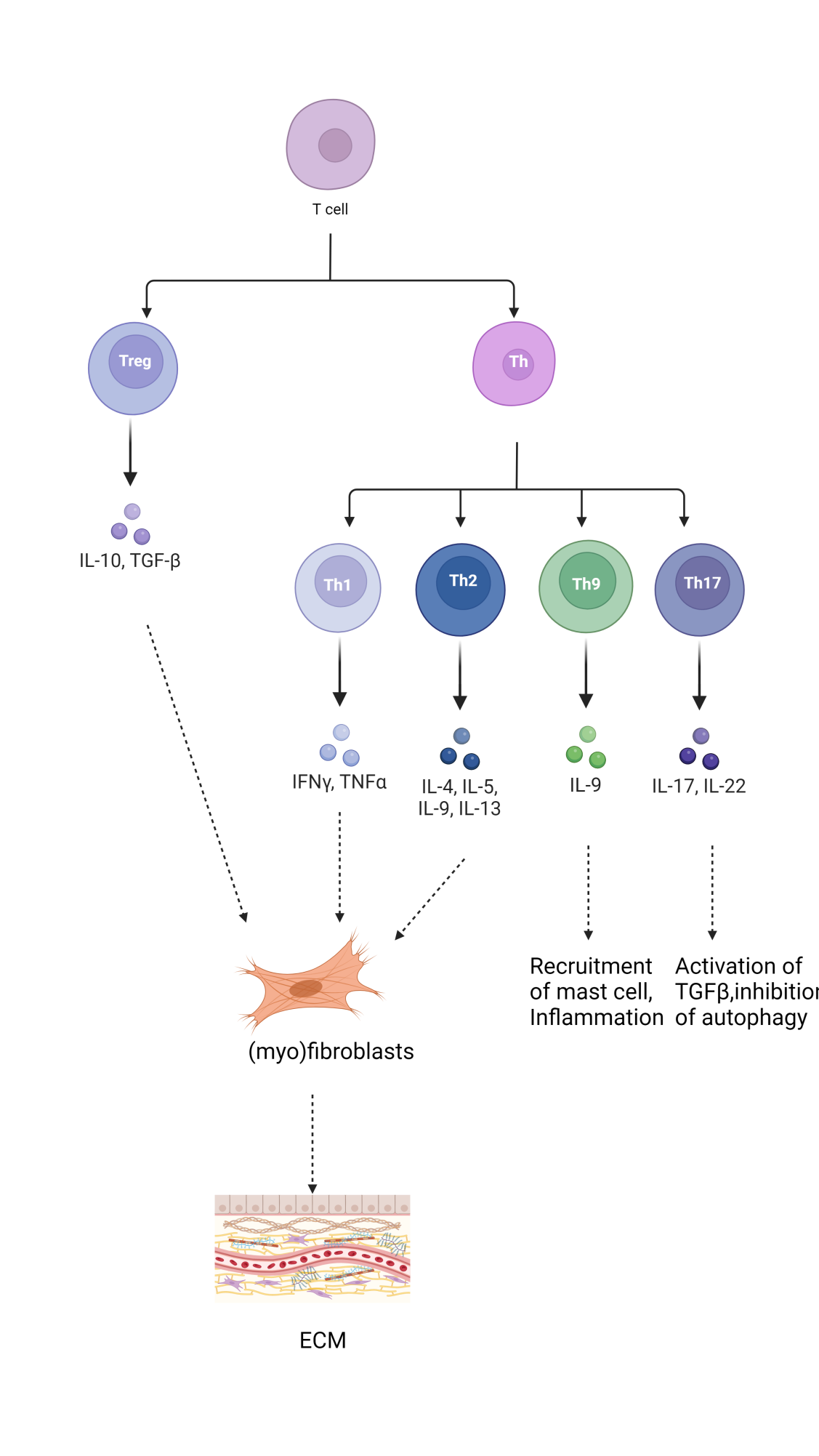
**

**Supplementary Figure 2.** Role of different T cells in pulmonary fibrosis.“+” represents “activation”;“-”represents “inhibition”.

**
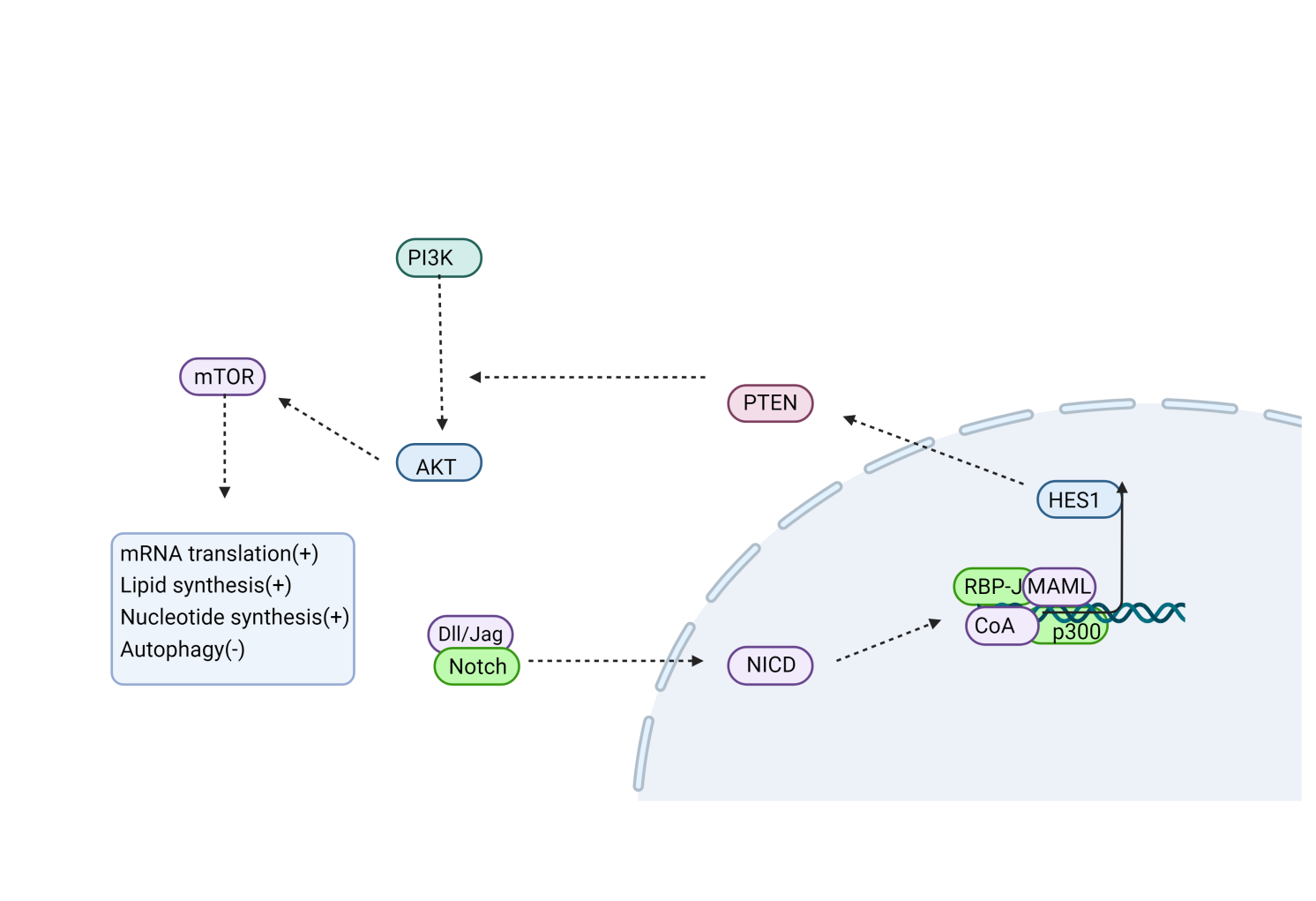
**

**Supplementary Figure 3.** Notch signaling is involved in regulating autophagy.NOTCH down-regulates PTEN protein expression via its downstream target gene HES1. HES1 subsequently activates PI3K, which in turn phosphorylates AKt, thereby transmitting signals to various downstream effectors including mTOR. Phosphorylated mTOR further phosphorylates downstream effector molecules, thus promoting protein synthesis and inhibiting autophagy.CoA, coactivator; MAML, Mastermind-like; “+” represents “activation”; “-”represents “inhibition”.
